# The impact of resistance training on gluteus maximus hypertrophy: a systematic review and meta-analysis

**DOI:** 10.3389/fphys.2025.1542334

**Published:** 2025-04-10

**Authors:** Walter Krause Neto, Thaís Lima Vieira Krause, Eliane Florencio Gama

**Affiliations:** ^1^ Department of Morphology and Genetics, Federal University of São Paulo, São Paulo, São Paulo, Brazil; ^2^ Military Fire Brigade Group of the Federal District, Brasilia, DF, Brazil

**Keywords:** resistance training, skeletal muscle, hip, muscle mass, exercise

## Abstract

This systematic review aims to examine and synthesize the existing literature regarding gluteus maximus (GMax) hypertrophy as a result of dynamic exercises that incorporate hip extension with external resistance. A comprehensive search was undertaken across the following databases: PubMed/Medline, SportDiscuss, Scopus, Web of Science, CINAHL, Science Direct, Google Scholar, and ResearchGate. Twelve articles met the established inclusion criteria, leading to the subsequent key findings: 1) resistance training exhibits a moderate effect on GMax hypertrophy (11 studies, SMD 0.71, 95% CI [0.50, 0.91], p < 0.00001, I^2^ = 22%); 2) subgroup analyses of single (seven studies, SMD 0.74, 95% CI [0.36, 1.13], p = 0.0001, I^2^ = 52%) and combined (six studies, SMD 0.68, 95% CI [0.44, 0.92], p < 0.00001, I^2^ = 0%) training protocols have demonstrated moderate effects; 3) when emphasizing GMax hypertrophy at the expense of other muscle groups, single exercises, such as the barbell hip thrust, should be prioritized; 4) back squats performed in parallel or full range of motion significantly enhance GMax hypertrophy; 5) leg press machines and kneeling hip extensions can also facilitate increased GMax hypertrophy; 6) training programs that incorporate combined hip extension exercises, whether single-joint or multi-joint, significantly promote an increase in GMax hypertrophy. This study concludes that a variety of exercises—whether focused on a specific joint (single-joint) or encompassing multiple joints (multi-joint)—can effectively stimulate GMax hypertrophy, whether executed individually or in combination.

## Introduction

The gluteus maximus (GMax) represents the largest and most powerful muscle in the gluteal region, playing a significant role in terms of strength, movement and functionality (Stern, 1972; Barker et al., 2014; Hogervorst and Vereecke, 2014; Williams et al., 2021; Duke et al., 2025).

Anatomically, the GMax receives its innervation from the inferior gluteal nerve (McCrory and Bell, 1999). The GMax muscle originates from the sacrum, coccyx, sacrotuberous ligament, ilium, thoracolumbar fascia, and gluteal aponeurosis, as indicated by Barker et al. (2014). Its fibers extend inferolaterally towards the femur; the superficial fibers contribute to the formation of a tendinous lamina that is part of the iliotibial tract. Meanwhile, the deeper fibers insert into the gluteal tuberosity of the femur, as noted by Stern (1972) and Barker et al. (2014). Furthermore, Takahashi et al. (2025) identified that the specific torque for hip extension and external rotation was generally more pronounced in the distal region compared to other areas. In contrast, the specific torque for hip abduction was found to be more substantial in the proximal region than in other regions. It is particularly noteworthy that the distal-lateral region exhibited a negative specific torque for hip abduction, suggesting that the fascicles in this specific area serve to facilitate hip adduction.

From a functional perspective, the GMax plays a role in movements across all planes of motion (Hoch et al., 2025; Takahashi et al., 2025). Consequently, alterations in the morphological or functional characteristics associated with dysfunctions of the pelvic muscles, including the GMax, may result in impaired physical functionality and contribute to the development of a degenerative pathological condition. According to Isawa et al. (2025), patients diagnosed with hip osteoarthritis showed significant muscle atrophy and fatty degeneration in the affected areas of the pelvis and thigh. Additionally, the volume of the GMax was associated with early postoperative physical function. Therefore, preoperative rehabilitation that targets gluteal muscle hypertrophy on the affected side may enhance recovery of physical function during the early postoperative period.

Hip extension is a crucial movement in everyday activities and sports. Research indicates that the role of hip extensor muscles increases during heavier lower-body exercises and in explosive sports, such as jumping and sprinting (Beardsley and Contreras, 2014; Loturco et al., 2018). While multiple muscles play a role in hip extension, the GMax is the primary muscle activated during activities that involve resistance or load, such as squats or hip thrusts (Krause Neto et al., 2019; Krause Neto et al., 2020; McCurdy et al., 2018; Williams et al., 2021). These exercises primarily target the GMax while minimizing the involvement of the hamstrings (Kubo et al., 2019; Plotkin et al., 2023). The GMax is essential for generating powerful hip extension, mainly when there is a high demand for strength, and stabilizing the pelvis during these movements is crucial. Understanding this helps in optimizing workout routines for strength training and rehabilitation.

The morphology of the GMax is associated with enhanced performance in athletes across various proficiency levels. Miller et al. (2021) underscore the significant differences in muscle composition observed across multiple anatomical regions, which are crucial for optimal sprinting capabilities. Research indicates that larger volumes of the hip extensors and GMax muscles effectively distinguish elite sprinters from their sub-elite counterparts, demonstrating a strong correlation with sprint performance. Takahashi et al. (2022) conducted a study emphasizing the differences in muscle structure between sprinters and other athletic groups, with a particular focus on the GMax. Their findings indicated that sprinters exhibit a greater volume in the distal regions of the GMax than individuals who do not engage in sprinting. This anatomical variation is attributable to the specific demands of sprinting, primarily enacted in the sagittal plane. This plane’s predominant nature of movement necessitates enhanced strength and power from the GMax, prompting its adaptation and development among sprinters. This suggests that the distinct biomechanical requirements of specialized physical exercises may significantly contribute to the observed morphological characteristics inherent in the muscle’s structure.

Resistance training is widely recognized as a crucial component of structured physical activity. It is particularly efficacious in eliciting substantial modifications in muscle architecture, a phenomenon referred to as muscle hypertrophy. As detailed in the systematic review conducted by Krause Neto et al. (2020), there exists considerable interest in determining the most effective exercises for enhancing GMax hypertrophy, motivated by both aesthetic considerations and enhancements in function and performance.

In recent years, a growing body of research has highlighted the effectiveness of various strength exercises in promoting hypertrophy in the GMax. Noteworthy isolated strength exercises that have been examined include leg presses (Popov et al., 2006) and back squats (Kubo et al., 2019), which engage multiple muscle groups and primarily target the lower body; barbell hip thrusts (Plotkin et al., 2023), which specifically concentrate on hip extension and effectively activate the glutes; and kneeling kickbacks (Ekechukwu and Okoh, 2020), which highlight glute engagement through an alternative movement pattern.

However, the research surrounding GMax muscle hypertrophy reveals significant methodological shortcomings, including limited sample sizes, a lack of technical standardization, and inconsistent methodologies. For instance, the study conducted by Barbalho et al. (2020) compared GMax thickness before and after a 12-week training period between two groups of trained women engaged in full-back squats (with 140° knee flexion) and barbell hip thrust exercises; however, it provided an insufficient description of the technique within the methodology section. It is essential to acknowledge that various methodological deficiencies may have impacted the outcomes, including the lack of data on prior training volume, inadequate descriptions of exercise movement techniques, and inconsistent strength gain values compared to existing literature, among other complications. Furthermore, Ekechukwu and Okoh (2020) explored the effects of donkey kicks (kneeling kickbacks) and squats in a sample of one hundred and seven women. Nevertheless, the authors did not employ an imaging method for assessment, which diminishes the quality and impact of the findings. These factors accentuate a specific gap within literature.

Given the abundance of information regarding various exercises, a significant inquiry emerges: What are the most effective strength exercises specifically designed to induce hypertrophy in the GMax? This inquiry constitutes the central focus of our systematic review, which aims to provide evidence-based recommendations for effective training regimens that facilitate GMax hypertrophy.

To address this question, this systematic review thoroughly examined and summarized the existing literature on GMax hypertrophy resulting from dynamic exercises that feature hip extension combined with external resistance. This review provided a comprehensive overview of the exercises studied and their effectiveness in promoting muscle growth in GMax. It also offered practical implications for fitness professionals and individuals in selecting the most beneficial training regimens for achieving gluteal hypertrophy.

## Methods

To conduct a systematic review, we used the Preferred Reporting Items for Systematic Reviews and Meta-Analyses (PRISMA) statement guide (Moher et al., 2009).

### Search strategy

From October 28th to 04 November 2024, a systematic search was conducted using PubMed/Medline, SportDiscuss, Scopus, Web of Science, CINAHL, and Science Direct electronic databases. Further, we used Google Scholar and Research Gate to search manually for any additional articles that could meet the inclusion criteria. Starting from these dates, any new research has been evaluated and included if it meets the established inclusion criteria. The MeSH descriptors, along with the related terms and keywords, were used as follows (resistance training OR resistance exercise OR training, resistance OR strength training OR training, strength OR weight-lifting strengthening program OR strengthening program, weight-lifting OR strengthening programs, weight-lifting OR weight lifting strengthening program OR weight-lifting strengthening programs OR weight-lifting exercise program OR exercise program, weight-lifting OR exercise programs, weight-lifting OR weight lifting exercise program OR weight-lifting exercise programs OR weight-bearing strengthening program OR strengthening program, weight-bearing OR strengthening programs, weight-bearing OR weight bearing strengthening program OR weight-bearing strengthening programs OR weight-bearing exercise program OR exercise program, weight-bearing OR exercise programs, weight-bearing OR weight-bearing exercise program OR weight-bearing exercise programs OR isometric OR exercise OR rehab OR physical therapy OR load OR training) AND (muscle development OR development, muscle OR muscular development OR development, muscular OR myogenesis OR myofibrillogenesis OR muscle hypertrophy OR hypertrophy OR hypertrophies) AND (gluteus maximus OR gluteus OR hip extensor OR hip extensors). Additionally, we reviewed the reference lists of the retrieved articles to identify supplementary studies that may fulfill the inclusion criteria.

### Inclusion and exclusion criteria

Studies meeting the following criteria were included in this review: (a) original articles, irrespective of whether they involved randomization; (b) investigations assessing alterations in GMax morphology; (c) training protocols extending for a minimum duration of 5 weeks; (d) comparisons of intra-group results utilizing pre- and post-intervention measurements; (e) publications available in the English language; and (f) studies involving healthy human participants aged between 18 and 45 years. Regarding muscle hypertrophy outcomes, only studies that evaluated any of the following metrics were considered: muscle thickness, muscle cross-sectional area, or GMax muscle volume assessed via ultrasound or magnetic resonance imaging (MRI). Excluded from this review were studies that presented inadequate data, review articles, conference papers, student theses, samples from metabolic patients, individuals with musculoskeletal trauma, elderly participants, poorly executed data presentations, and protocols that were unclear or vague. In instances where the articles had the potential for inclusion but lacked sufficient data, we reached out to the authors via email to request additional information.

### Studies selection and quality assessment

The authors, WKN, TLVK, and EFG, independently conducted the data analysis, followed by two meetings to determine the eligibility of articles for inclusion in the final manuscript. Each reviewer meticulously managed duplicate records. During the initial meeting, the researchers compared their search results and agreed on the articles that warranted full-text analysis. The outcomes reflected in the tables were reviewed in the subsequent meeting, determining the final articles to be included in the systematic review and meta-analysis. After each article was examined, the following data were extracted: sample size, group randomization, assessment of blinding outcomes, experience with resistance training, training protocol, duration of training, hypertrophic outcomes, diagnostic methods, summary statistics, and main hypertrophic findings.

After reading the titles and abstracts and deciding on full-text inclusion, all eligible studies’ quality was assessed using the Downs and Black assessment tool (Downs and Black, 1998). This questionnaire is a checklist containing 27 items that address the following aspects of study design: reporting (items 1–10), external validity (items 11–13), internal validity (items 14–26), and statistical power (item 27). Following the suggestion of Coleman et al. (2022) and others (Grgic et al., 2018), we modified the checklist by adding two items related to participant adherence (item 28) and training supervision (item 29). The scoring of each item in the checklist proceeds as follows: one point is given (+1) if the criterion is met or zero (0) if the criterion is not met. The studies were then classified as: “good quality” (21–29 points), “moderate quality” (11–20 points), or “low quality” (less than 11 points) (Grgic et al., 2020).

### Data synthesis and meta-analysis

The systematic review data are presented in Tables 1, 2. The mean values and standard deviations were extracted from each study and used for meta-analysis calculations. If the studies did not show all the data necessary for meta-analysis, the corresponding authors were emailed requesting them. For statistical analysis, we used Review Manager software 5.4.1 to calculate the standardized mean difference [(SMD)] the mean of the PRE time points minus the mean of the POST time point divided by the pooled SD of the two groups), 95% confidence interval (95% CI), and heterogeneity by I^2^, Chi^2^, and Tau^2^ values. We used I^2^ to assess heterogeneity between studies using random-effect models (I^2^ values <50% indicate low heterogeneity, 50%–75% moderate heterogeneity, and >75% high heterogeneity). Subgroup analysis was applied as necessary. For the overall effect, p ≤ 0.05 was considered statistically significant. Risks of bias are presented in the forest plot graphic. Publication bias was assessed by analyzing the asymmetry of the funnel plot.

## Results

### Search results

**FIGURE 1:**
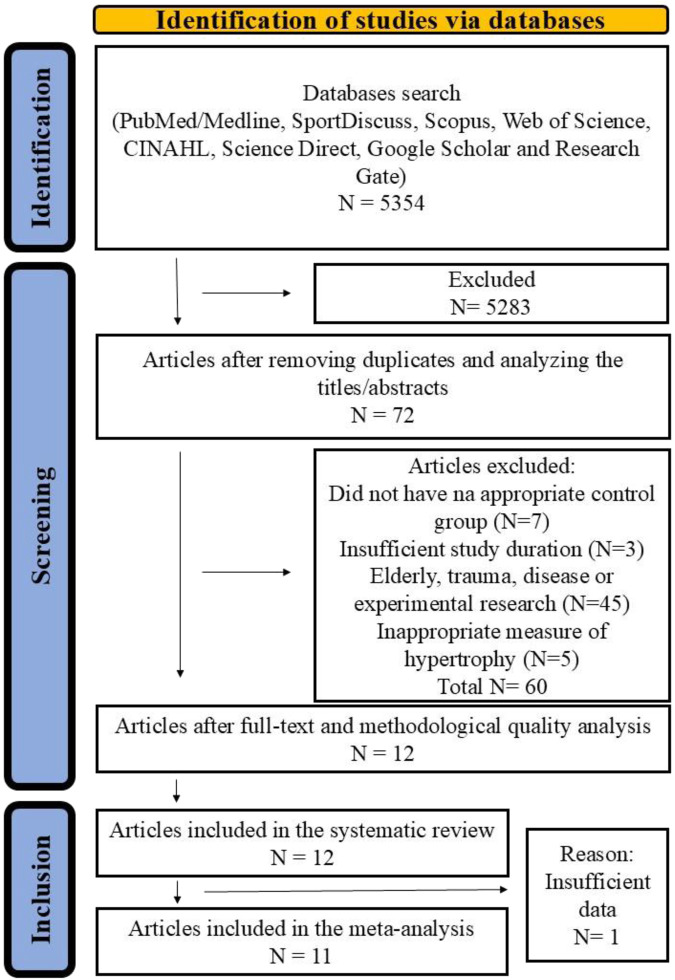
PRISMA flowchart corresponding to the article search process.

The initial survey identified 5,354 articles. After removing duplicates and analyzing the titles/abstracts, 5,283 articles were eliminated, leaving 72 articles selected for full-text analysis. Of these, 12 were considered eligible and included in this systematic review (Popov et al., 2006; Kubo et al., 2019; Trindade et al., 2019; Barbalho et al., 2020; Barbalho et al., 2021; Nakamura et al., 2021; Short et al., 2021; Balshaw et al., 2023; Plotkin et al., 2023; Wei et al., 2023; Kassiano et al., 2024; Bartolomei et al., 2024). Additionally, three studies were included in the meta-analysis after the authors sent us additional data via email (Kubo et al., 2019; Plotkin et al., 2023; Bartolomei et al., 2024). However, one study did not have enough statistical information and was not included in the meta-analysis (Short et al., 2021). Figure 1 presents the PRISMA flow chart of the complete search process.

### Methodological quality

The qualitative assessment of the studies showed a mean score of 19.5 points (moderate, range: 13 to 23 points). Based on the study quality categorization criteria, six studies were good quality (Kubo et al., 2019; Trindade et al., 2019; Nakamura et al., 2021; Short et al., 2021; Balshaw et al., 2023; Wei et al., 2023), and six were considered moderate quality (Popov et al., 2006; Barbalho et al., 2020; Barbalho et al., 2021; Plotkin et al., 2023; Kassiano et al., 2024; Bartolomei et al., 2024). No study included in this systematic review was classified as low quality.

**FIGURE 2:**
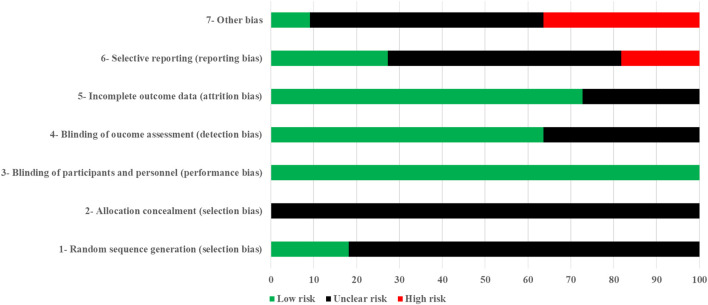
Risk of bias averaged per question.

**FIGURE 3:**
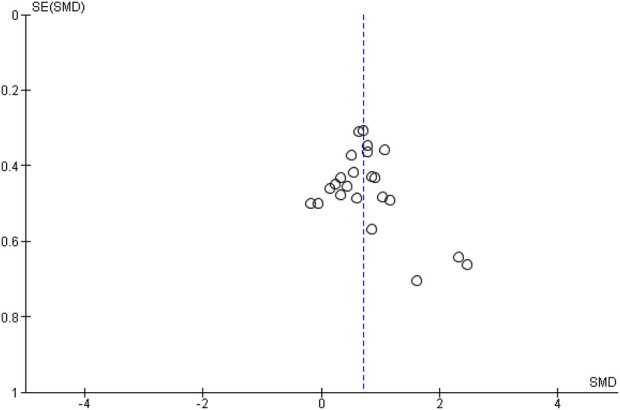
Funnel plot of standardized mean differences (SMD) of gluteus maximus hypertrophy. SE = standard error.

Figure 2 illustrates the average results of the risk of bias assessment. Overall, random sequence generation showed a low risk of bias in 18.18% of the studies. In contrast, all studies exhibited an unclear risk of bias for allocation concealment. For the blinding of participants and personnel, we assessed all studies as having a low risk of bias since it is not feasible to blind participants or coaches in sports science studies. Regarding the detection of outcome assessment, 63.64% of the studies were evaluated as having a low risk of bias, while 36.36% were deemed to have an unclear risk. Incomplete outcome data was rated as having a low risk of bias in 72.73% of the studies. Selective reporting was classified as low, ambiguous, and high risk of bias in 27.27%, 54.55%, and 18.18% of studies. Finally, other types of bias were classified as unclear or high risk in 90.91% of the studies. The lack of a complete technical description of the exercises negatively impacts the reproducibility of the research method. Funnel plots displayed normal symmetry (Figure 3).

### Resistance training protocol characteristics

In total, 318 participants (144 women and 174 men) underwent 11 variations of traditional strength exercises with external resistance, ten variations of bodyweight squats, and two variations of jumps. Eight studies used untrained or detrained participants (Popov et al., 2006; Trindade et al., 2019; Kubo et al., 2019; Nakamura et al., 2021; Balshaw et al., 2023; Plotkin et al., 2023; Wei et al., 2023; Kassiano et al., 2024), while four studies used trained individuals (Barbalho et al., 2020; Barbalho et al., 2021; Short et al., 2021; Bartolomei et al., 2024). All studies were randomly divided into groups, except for Popov et al. (2006). However, no study described the form and criteria used in randomization. Six studies attempted to blind those measuring the primary outcomes of the intervention (Trindade et al., 2019; Barbalho et al., 2020; Barbalho et al., 2021; Balshaw et al., 2023; Wei et al., 2023; Kassiano et al., 2024). Eight studies used ultrasound (Trindade et al., 2019; Barbalho et al., 2020; Barbalho et al., 2021; Nakamura et al., 2021; Short et al., 2021; Wei et al., 2023; Kassiano et al., 2024; Bartolomei et al., 2024) and four used magnetic resonance (Popov et al., 2006; Kubo et al., 2019; Balshaw et al., 2023; Plotkin et al., 2023). Eight studies measured muscle thickness (Trindade et al., 2019; Barbalho et al., 2020; Barbalho et al., 2021; Nakamura et al., 2021; Short et al., 2021; Wei et al., 2023; Kassiano et al., 2024; Bartolomei et al., 2024), three measured muscle volume (Popov et al., 2006; Kubo et al., 2019; Balshaw et al., 2023), and one measured cross-sectional area (Plotkin et al., 2023). Most articles quoted the area of ultrasound measurement as being done in the cranial (upper) GMax. Only the study of Plotkin et al. (2023) measures the GMax in three different places: the low, middle, and upper glutes. Table 1 describes studies according to participants’ number, training status, group randomization, hypertrophic outcomes, and diagnostic method.

**TABLE 1:** The descriptions of the studies were included according to participants’ numbers, training status, group randomization, blinding outcome assessment, hypertrophic assessment, summary statistics and diagnostic method.

References	Participants number	Training status	Groups randomization	Blinding outcome assessment	Hypertrophic assessment	Diagnostic method
Popov et al. (2006)	18 male (21 ± 2 years)	Untrained	Not mentioned	Not mentioned	Muscle volume Not detailed about the measurement position	Magnetic resonance
Trindade et al. (2019)	31 males (31.37 ± 6.83 years-old)	Detrained (at least 1 year of training experience but not in the 6 months before the study)	Yes	Yes	Muscle thickness (50% of the sacral vertebra, and the greater trochanter of the femur) Prone position	Ultrasound
Kubo et al. (2019)	17 males	Untrained	Yes	Not mentioned	Muscle volume (Transverse scans were performed from the iliac crest to gluteal tuberosity) Prone position	Magnetic resonance
Barbalho et al. (2020)	22 women (BS = 26.4 ± 1.3 years-old and HT = f 27.5 ± 1.4 years-old)	Trained (BS = 4.8 ± 0.8 years and HT = 5.1 ± 0.7 years of experience)	Yes	Yes	Muscle thickness (50% of the distance between the sacral vertebra and the greater trochanter) Not detailed about the measurement position	Ultrasound
Barbalho et al. (2021)	30 women (MJ = 28.2 ± 1.67; SJ = 29.4 ± 1.56 MJ + SJ = 28.6 ± 1.42 years-old)	Trained (MJ = 5.5 ± 0.84 SJ = 5.5 ± 0.97; MJ + SJ = 5.2 ± 0.91 years of experience)	Yes	Yes	Muscle thickness (50% of the distance between the sacral vertebra and the greater trochanter) Not detailed about the measurement position	Ultrasound
Nakamura et al. (2021)	16 males (21.3 ± 1.1 years old)	Untrained	Yes	Not mentioned	Muscle thickness (30% proximal to between the posterior superior iliac spine and the greater trochanter) Prone position	Ultrasound
Short et al. (2021)	46 participants (17 males, 29 females) (mean age 22.5 ± 2.3 years old)	Intermediate trained (healthy individuals who participated in exercise training within the past 3 months)	Yes	Not mentioned	Muscle thickness (the image was found by gliding up the hamstrings until the ischial tuberosity was identified) Prone position	Ultrasound
Balshaw et al. (2023)	38 male [CP (n = 19) or PLA (n = 20)]	Untrained	Yes	Yes	Muscle volume Supine position	Magnetic resonance
Plotkin et al. (2023)	34 participants (18 HT (5 male and 13 female, 22 ± 3 years old) and 16 SQ (6 male and 10 female, 24 ± 4 years old)	Untrained	Yes	Yes	Muscle cross-sectional area (mCSA) (a) the middle of the gluteus maximus was standardized at the image revealing the top of the femur, (b) the image that was ten slices upward from this mark was the upper gluteus maximus, (c) the image that was 18 slices downward from the top of the femur was considered lower gluteus maximus Prone position	Magnetic resonance
Wei et al. (2023)	13 women (19.77 ± 0.83 years)	Untrained	Yes	Yes	Muscle thickness (the first third between the posterior superior iliac spine and the greater trochanter of the femur) Lying or sitting in a relaxed position	Ultrasound
Kassiano et al. (2024)	33 women [L-S, n = 15 (22.6 ± 3.7 years); L-S-BHT, n = 18 [22.3 ± 4.4 years]	Untrained	Yes	Yes	Muscle thickness (50% of the distance between the sacral vertebra and the greater trochanter of the femur) Prone position	Ultrasound
Bartolomei et al. (2024)	19 participants [15 men and four women (2 in each group) were randomly assigned to the VT group (N. = 9; age: 26.9 ± 4.4) or to the HT group (N. = 10; age: 25.1 ± 4.0 years]	Trained (mean experience 7.6 ± 6.0 years)	Yes	Not mentioned	Muscle thickness (lower half of a line that connected the tip of the greater trochanter to the anterior quarter of a line that directly linked the anterior and posterior iliac spines) Not detailed about the measurement position	Ultrasound

*Legend: HT, hip thrust; SQ, squat; VT, vertical; HT, horizontal; L-S, leg press plus stiff; L-S-BHT, leg press plus stiff plus barbell hip thrust; MJ, multi-joint; SJ, single-joint; MJ + SJ, multi-joint plus single-joint.

Table 2 describes resistance training protocols, duration, and main findings. Nine studies investigated the effects of single exercises on GMax hypertrophy, such as leg press (Popov et al., 2006; Trindade et al., 2019; Balshaw et al., 2023), squat variations (Kubo et al., 2019; Barbalho et al., 2020; Nakamura et al., 2021; Plotkin et al., 2023; Wei et al., 2023), barbell hip thrust variations (Barbalho et al., 2020; Plotkin et al., 2023) and kneeling hip extension (Short et al., 2021). Moreover, five studies investigated the effects of combined multiple exercises (Trindade et al., 2019; Barbalho et al., 2021; Wei et al., 2023; Kassiano et al., 2024; Bartolomei et al., 2024). Six studies provided detailed descriptions of the exercise technique, including knee angles or illustrated images of the technique (Kubo et al., 2019; Nakamura et al., 2021; Short et al., 2021; Balshaw et al., 2023; Wei et al., 2023; Kassiano et al., 2024).

**TABLE 2:** Description of resistance training protocols, duration, and main findings.

References	Resistance training protocol	Duration	Main findings
Popov et al. (2006)	- Exercise: Leg press - Frequency: 3x/week - Groups - (I) Classic full-range strength training: one high-intensity session at 80% MVC, seven sets of 6–12 reps to failure (Monday), and two toning sessions of three sets (Wednesday and Friday) - (II) Continuous partial-range strength training: one low-intensity session at 50% MVC, four sets of 50–60 s with short rests (Monday), and two toning sessions with single sets (Wednesday and Friday)	8 weeks	Groups I and II increased significantly the gluteus maximus by 18% and 13%, respectively
Trindade et al. (2019)	- Exercises: Leg extension and Leg press 45° exercises - Frequency: 2x/week - Groups - Traditional: (TRT) Leg press with three sets of 75% 1-RM to failure - Pre-exhaustion: (PreEX) an additional set to failure on the leg extension (20% 1-RM) immediately before (≤10 s) the leg press set	9 weeks	Groups TRT and PreEX increased gluteus maximus percentage by 30% ± 44% and 25% ± 58%, respectively. However, neither was statistically significant
Kubo et al. (2019)	- Exercise: Back Squat - Frequency: 2x/week - Groups −140° knee flexion full squat (FS) −90° knee flexion half squat (HS) - From the first to fourth week, the subjects had three sets with increased intensity from 60% to 90%-1RM (8–10 reps). From the fifth week to the end, subjects performed three progressive sets of 8RMs loads	10 weeks	The volume of gluteus maximus significantly increased in FS and HS by 6.7% ± 3.5% and 2.2% ± 2.6%, respectively
Barbalho et al. (2020)	- Exercises: Back Squat and Barbell hip thrust - Frequency: 1x/week - Groups - Full range of motion (140°) back squat (BS) - Partial range of motion (started at 135° hip extension) barbell hip thrust (BHT) - Six sets per week - Non-linear periodization of repetitions to failure	12 weeks	Both groups significantly increased gluteus maximus thickness. However, the BS group (9.4%) had more significant gains than HT (3.7%)
Barbalho et al. (2021)	- Frequency: 1x/week - Groups - Multi-joint (MJ) = back squat, deadlift, lunge, and leg press - Single-joint (SJ) = barbell hip thrust and 4-point kneeling hip extension - MJ + SJ = back squat, barbell hip thrust, and 4-point kneeling hip extension - Training Volume: MJ = 12 sets, SJ = 5 sets, MJ + SJ = 8 sets - Non-linear periodization of repetitions to failure	24 weeks	Gluteus maximus thickness significantly increased in all groups. However, the MJ (14.5% approximately) and MJ + SJ (12.5% approximately) groups showed higher increases than SJ (3% approximately)
Nakamura et al. (2021)	- Exercise: Bilateral belt squat (90° knee flexion) using an inertial flywheel machine - Frequency: 2x/week - The training load was increased from a moment inertia of 0.025 kg m^2^ in the first session to 0.100 kg m^2^ in the 10th session. In each session, three sets of 10 repetitions of the exercise were performed with a 180-s rest between sets - Groups - Traditional (T) - Static stretching (ST) between sets	5 weeks	No changes
Short et al. (2021)	- Exercise: Kneeling hip extension with different tension bands - Frequency: 2x/week - Intra-subjects analysis - Groups - Traditional (TRAD) - Blood-flow restricted resistance training (BFR-RT) - Four sets of 75 repetitions (30, 15, 15, and 15 repetitions) were performed on each leg with a 30-s rest period between sets	6 weeks	Similar gluteus maximus thickness increases were found between groups. However, BFR-RT was clinically significant, resulting in clinically meaningful additional proximal muscle thickness
Balshaw et al. (2023)	- Exercises: Leg extension, leg flexion, and leg press 45 - Frequency: 3x/week - Groups - Collagen supplementation plus resistance training (CP) - Placebo plus resistance training (PLA) - Training volume: 2–4 sets of 6–12 RM.	15 weeks	There were similar increases between groups CP and PLA (16.6% vs. 12.9%), respectively
Plotkin et al. (2023)	- Exercises: Back Squat and Barbell hip thrust - Frequency: 2x/week - Groups - Barbell back squat (BS): as deep as possible - Scoop Barbell hip thrust (HT): Full range (80° approximately) - Training volume: Progressive 3–6 sets per session with 8–12 reps per set	9 weeks	Both interventions induced similar gluteus maximus cross-sectional area with more pronounced increases at the lower gluteus maximus
Wei et al. (2023)	- Exercises: Back Squat and bodyweight squat variations - Frequency: 2x/week - Groups - Progressive bodyweight squat (PB): 10 squat variations with volume weekly increases - Progressive barbell back Squat (BS) = 6 sets of 8–12 repetitions (60%–80% 1-RM)	6 weeks	Both interventions induced similar gluteus maximus thickness increases
Kassiano et al. (2024)	- Exercises: 45° leg press, stiff-leg deadlift, and barbell hip thrust - Frequency: 3x/week - Groups −45° leg press and stiff-leg deadlift (L-S) −45° leg press, stiff-leg deadlift, plus barbell hip thrust (L-S-BHT) - Training volume: Two sets of 10–15 repetitions per session	10 weeks	Both groups increased gluteus maximus thickness; however, group L-S-BHT presented more significant gains than L-S (9.3% and 6%, respectively)
Bartolomei et al. (2024)	- Exercises: Back squat, Barbell step-up, Barbell hip thrust, Reverse hyperextension, Box jump and long jump - Frequency: 2x/week - Groups - Vertical Training (VT): Back Squat, Barbell step-up and box jump - Horizontal Training (HT): Barbell hip thrust, reverse hyperextension, and long jump - Training volume: non-linear program	6 weeks	Both groups increased gluteus maximus thickness

Weekly training frequency varies between 1 and 3 sessions per week. Training volume varied between 3 and 12 sets per training session. Load intensity varied by % of 1-RM (50%–90% 1-RM) or repetition maximum (3–12RM). Although there was no time limit as an inclusion criterion, all the articles included in this review were published between 2006 and 2024.

### Main hypertrophic findings

In four studies, barbell back squats significantly increased GMax (Kubo et al., 2019; Barbalho et al., 2020; Plotkin et al., 2023; Wei et al., 2023). All four studies demonstrated that ROM matters for GMax hypertrophy; however, squatting till parallel should be enough (Plotkin et al., 2023; Wei et al., 2023).

One study showed that the flywheel squat machine did not change GMax (Nakamura et al., 2021).

In two studies, the barbell hip thrust exercise trained alone significantly increased GMax (Barbalho et al., 2020; Plotkin et al., 2023). Two studies also showed that the full ROM barbell hip thrust induced more significant GMax gains (Plotkin et al., 2023; Kassiano et al., 2024) than the short range of motion (Barbalho et al., 2020). Both barbell hip thrust scoop (Plotkin et al., 2023) and hinge (Kassiano et al., 2024) techniques can efficiently promote GMax hypertrophy.

One study showed that the leg press machine significantly increased GMax (Popov et al., 2006), while another did not (Trindade et al., 2019).

One study showed that the kneeling hip extension with increased tension bands significantly increased GMax (Short et al., 2021).

One study showed that bodyweight squat variations significantly increased GMax (Wei et al., 2023).

In three studies, combined multiple hip extension exercises, even with single or multi-joint exercises, increased GMax (Barbalho et al., 2021; Kassiano et al., 2024; Bartolomei et al., 2024). One study showed that combining single-joint exercises (knee extension and knee flexion exercises) with leg press did not negatively influence GMax increase (Balshaw et al., 2023). However, one study showed that the quadriceps pre-exhaustion method (with knee extension machine before leg press exercise) may negatively influence GMax increase (Trindade et al., 2019).

### Meta-analysis

**FIGURE 4:**
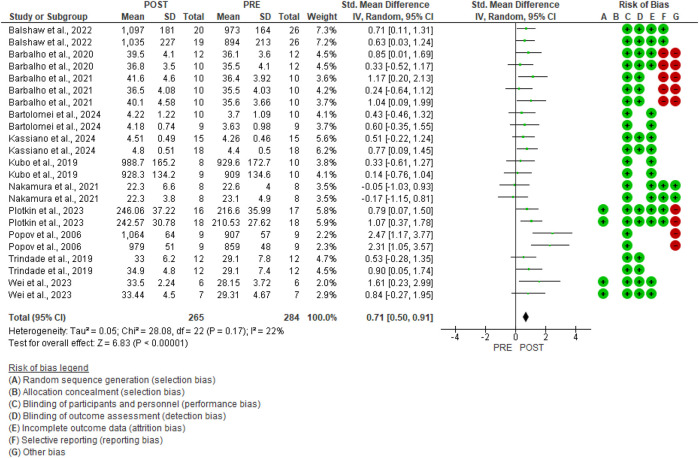
Forest plots of the data examining the effect of resistance training on gluteus maximus hypertrophy (produced in the Review Manager 5.4.1 software). The appearance of the same study multiple times indicates more than one exercise in the research.

GMax hypertrophy was confirmed by meta-analysis (11 studies, Figure 4). Figure 4 also presents the risk of bias for each study. Resistance training demonstrated a moderate effect [SMD 0.71, 95%CI (0.50, 0.91), p < 0.00001]. A low level of heterogeneity was found in the studies (p = 0.17, I^2^ = 22%).

**FIGURE 5:**
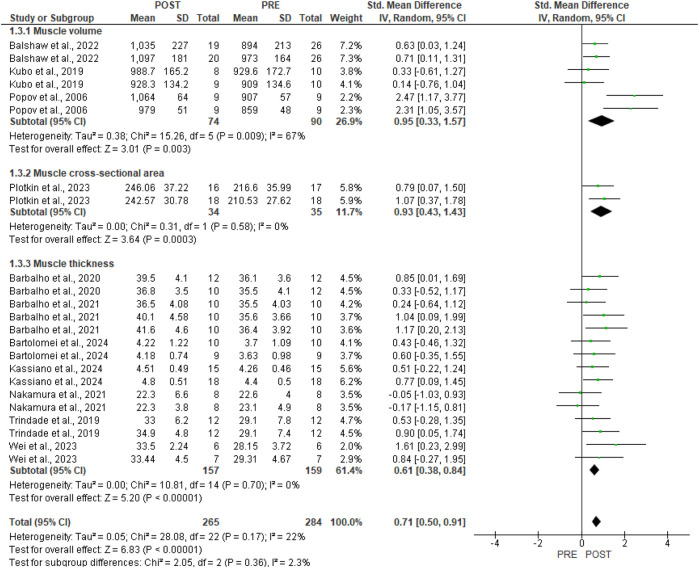
Forest plots of gluteus maximus hypertrophy were examined by the type of assessment (produced in the Review Manager 5.4.1 software). The appearance of the same study multiple times indicates more than one exercise in the research.

In Figure 5, sub-group analysis for the type of hypertrophic outcome reveals a large effect for muscle volume [three studies, SMD 0.95, 95%CI (0.33, 1.57), p = 0.003, I^2^ = 67%] and cross-sectional area [one study, SMD 0.93, 95%CI (0.43, 1.43), p = 0.0003, I^2^ = 0%]. Muscle thickness assessment presented a moderate effect [seven studies, SMD 0.61, 95%CI (0.38, 0.84), p < 0.00001, I^2^ = 0%].

**FIGURE 6:**
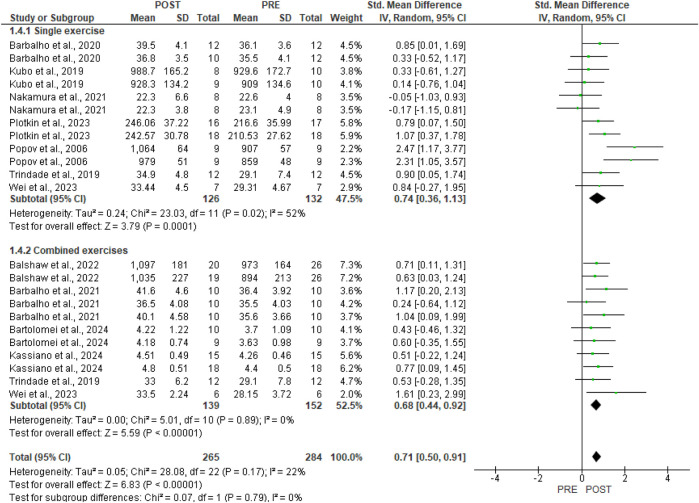
Forest plots of gluteus maximus hypertrophy were examined by the effect of isolated and combined exercise protocols (produced in the Review Manager 5.4.1 software). The appearance of the same study multiple times indicates more than one exercise in the research.

In Figure 6, sub-group analysis for the single [seven studies, SMD 0.74, 95%CI (0.36, 1.13), p = 0.0001, I^2^ = 52%] and combined [six studies, SMD 0.68, 95%CI (0.44, 0.92), p < 0.00001, I^2^ = 0%] training protocols demonstrated moderate effects.

The substantial heterogeneity observed in the muscle volume outcome (I^2^ = 67%) and the single exercise outcome (I^2^ = 52%) seems to have been affected by the larger effect size reported by Popov et al. (2006).

**FIGURE 7:**
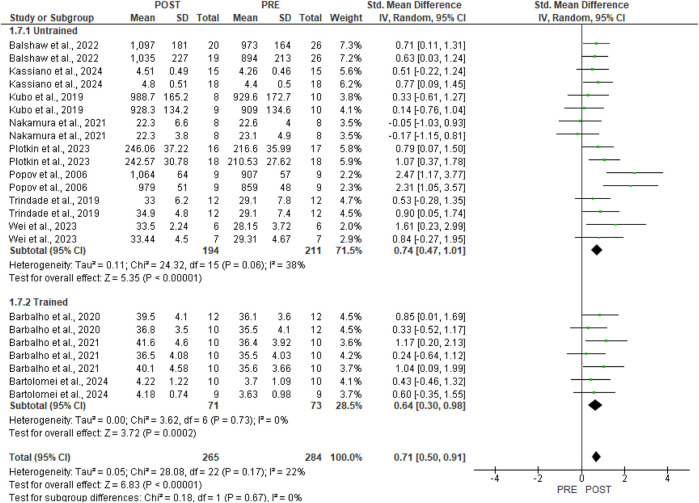
Forest plots of gluteus maximus hypertrophy were examined by the effects of the training status (produced in the Review Manager 5.4.1 software). The appearance of the same study multiple times indicates more than one exercise in the research.

In Figure 7, sub-group analysis for the untrained [eight studies, SMD 0.74, 95%CI (0.47, 1.01), p < 0.00001, I^2^ = 38%] and trained [three studies, SMD 0.64, 95%CI (0.30, 0.98), p = 0.0002, I^2^ = 0%] training status demonstrated moderate effects.

## Discussion

This systematic review, coupled with a meta-analysis, revealed significant diversity in exercise modalities—both individual and combined—that effectively promote hypertrophy of the GMax muscle. The review highlights the importance of selecting exercises that align with individual fitness levels and goals. It suggests that a well-rounded program incorporating a variety of exercises may yield the best results for GMax hypertrophy.

Among the various multi-joint exercises available, the back squat is the most widely recognized strength exercise for fostering muscle hypertrophy, particularly in the lower limbs. In 2019, a study by Kubo et al. (2019) shed light on the efficacy of the back squat, presenting compelling evidence that it significantly enhances the volume of the GMax muscle in untrained individuals.

The researchers underscored a critical factor associated with the hypertrophy of the GMax: the range of motion (ROM) during the exercise. The findings of Kubo et al. (2019) illustrated that an increased ROM during the back squat is correlated with more substantial gains in GMax volume. Individuals who perform back squats with deeper and more extensive motion tend to experience greater muscular adaptations than those who restrict their movement to 90 degrees of knee flexion. Therefore, optimizing the ROM in back squats is essential for maximizing the benefits of this foundational exercise in GMax training. Supporting these conclusions, Barbalho et al. (2020) found that a back squat executed with 140° of knee flexion (full ROM) significantly enhances the thickness of the GMax in experienced women (with a mean of 5 years of experience) following 3 months of strength training.

Not all practitioners can achieve an extended ROM, as noted by Kubo et al. (2019) and Barbalho et al. (2020), due to physical limitations or insufficient joint mobility. Wei et al. (2023) found that performing back squats to a parallel ROM significantly increases the thickness of the GMax. Additionally, Plotkin et al. (2023) demonstrated that individuals who performed back squats with the maximum attainable ROM exhibited a significant increase in the cross-sectional area of the GMax. The authors also suggested that maximum ROM should be customized for each individual. Supplementary information provided by the author reveals that the majority of subjects engaged in training with a range of motion ROM at or below the parallel line of the femur in relation to the ground. Specifically, five volunteers conducted their training below parallel, ten volunteers trained at a position marginally above or below parallel, and only one subject executed a half squat, characterized by approximately 90° of knee flexion. Although the authors allowed a range of motion, the results show that the hypertrophic response from squatting was significant. This suggests that it is more crucial for each trainee to work within their limits rather than adhere to a one-size-fits-all approach to maximum ROM. This information is vital not only for aesthetic enhancements but also in physical rehabilitation settings, where ROM often tends to be more restricted.

Nakamura et al. (2021) investigated the effects of the flywheel bilateral belt squat exercise on the thickness of the GMax. The findings indicated that executing the flywheel squat exercise with a knee flexion angle of up to 90° did not significantly alter the thickness of the GMax. These results imply that not all variations of squats are effective in modifying the morphology of the GMax. Indeed, only parallel and full-range barbell back squats have been shown to foster GMax growth in healthy individuals effectively.

The squat represents an exercise that can be performed through various technical variations, including lunges, Bulgarian split squats, and single-leg squats. Wei et al. (2023) demonstrated that progressive resistance training utilizing bodyweight squat variations significantly contributes to hypertrophic gains in the GMax that are comparable to those achieved through traditional and intensive barbell back squat training among previously sedentary women. These findings suggest that integrating bodyweight strength training with progressive volume and squat variations may present a promising approach for enhancing GMax hypertrophy in untrained women. Furthermore, the research methodology implemented by Wei et al. (2023) suggests that resistance training using body weight can be effectively initiated in home and rehabilitation settings, as the increase in GMax thickness observed was similar to that found in the cohort that underwent high-intensity barbell back squat training.

The leg press apparatus is a prevalent component in fitness facilities worldwide, primarily used for targeting multiple joints and muscle groups. Leg press exercises primarily target the knee and hip extensor muscles, including the quadriceps femoris and GMax. Research conducted by Popov et al. (2006) indicated that executing the leg press across its full ROM, which encompasses complete extension and flexion of the knees, can result in hypertrophy of the GMax, comparable to that attained through partial range training.

Moreover, a study conducted by Balshaw et al. (2023) found that leg press training facilitates hypertrophy of the GMax, regardless of whether supplementary exercises such as leg extensions or knee flexions are included in the workout regimen. Conversely, Trindade et al. (2019) observed no significant alterations in GMax thickness after the training, thereby underscoring the discrepancies in findings across various studies.

The variations observed in this study regarding the hypertrophic muscle response during leg press exercises can be attributed to several factors, including the methodologies employed for outcome assessment and the technical aspects associated with the exercise’s execution. Firstly, Popov et al. (2006) and Balshaw et al. (2023) utilized magnetic resonance imaging to evaluate GMax volume, whereas Trindade et al. (2019) employed ultrasound to assess GMax thickness. According to Haun et al. (2019), the methodology for assessing muscle morphology may influence outcomes due to discrepancies in intramuscular and intracellular content. Secondly, the type of leg press equipment used can significantly impact exercise results, as different machines alter the biomechanics involved in the movement, leading to variations in muscle engagement (Trindade et al., 2019; Kinoshita et al., 2024). Notably, the studies conducted by Trindade et al. (2019) and Balshaw et al. (2023) employed a leg press set at a 45-degree incline, while Popov et al. (2006) did not specify the type of equipment used. Furthermore, the ROM in leg press exercises holds significant importance during hip extension. The investigation by Trindade et al. (2019) appears to have employed a fixed ROM of 90 degrees of knee flexion. Consequently, engaging in leg press exercises with an enhanced ROM may yield more substantial changes in GMax hypertrophy, suggesting that variations in displacement distance may effectively target different muscle groups. Moreover, foot positioning techniques contribute to variations in muscle activation (Da Silva et al., 2008). For instance, the angle and positioning of the feet on the platform may redirect focus toward distinct muscle groups in the hips and legs. Recent research by Kinoshita et al. (2024), presented at the 29th Annual Congress of the European College of Sports Sciences, emphasized the benefits of unilateral training utilizing a horizontal leg press machine. The findings revealed that positioning the foot on the top of the platform significantly enhances GMax volume compared to the hypertrophy of other muscles in the hip and lower limb regions. This observation suggests that this specific foot positioning may enhance the efficacy of leg press training, particularly in targeting the GMax while minimizing hypertrophy in other muscle groups.

It is essential to emphasize that squat and leg press exercises are crucial for promoting hypertrophic gains across various muscle groups, particularly the quadriceps femoris (Trindade et al., 2019; Kubo et al., 2019; Barbalho et al., 2020; Plotkin et al., 2023). Therefore, in specific cases—particularly for female bodybuilding athletes competing in the Wellness and Bikini categories, where significant quadriceps development must be carefully regulated—the squat and leg press may not be the most effective exercises for enhancing hypertrophy in the GMax. Consequently, the selection of exercises that optimally stimulate GMax hypertrophy should be guided by the unique characteristics of each practitioner. Furthermore, patients with orthopedic conditions, such as osteoarthritis or hip arthrosis, often struggle to strengthen the GMax using squats and leg presses, as well as their variations.

In the quest to optimize athletic, aesthetic, and functional performance, it is crucial to prioritize exercises that specifically target the GMax, even if this focus may have only a marginal impact on other muscle groups. Recently, the barbell hip thrust has gained significant attention from both the scientific community and fitness professionals due to its unique mechanical properties and the considerable neuromuscular demands it places on the hip extensor muscles (Contreras et al., 2011; Dello Iacono et al., 2018; Dello Iacono and Seitz, 2018; Loturco et al., 2018; Williams et al., 2021). This exercise produces activation profiles that differ from those seen in traditional movements such as squats (including both front and back barbell variations), split squats, and deadlifts (Andersen et al., 2018; Williams et al., 2021). The barbell hip thrust requires engagement from the knee, hip, and pelvic-trunk joints, primarily activating the musculature of the hip extensors (Brazil et al., 2021). In 2020, Barbalho et al. demonstrated that the barbell hip thrust significantly boosts the hypertrophy of the GMax. Additionally, Plotkin et al. (2023) found that the barbell hip thrust promotes hypertrophy of the GMax without significantly increasing the cross-sectional area of the hamstrings, hip adductors, or quadriceps femoris. Finally, Kassiano et al. (2024) demonstrated that incorporating the barbell hip thrust into a training regimen alongside other multi-joint exercises explicitly enhances the thickness of the GMax. These findings suggest that exercises such as the barbell hip thrust are preferable to multi-joint exercises like back squats when the practitioner’s goal is to prioritize hypertrophy of the GMax. Furthermore, understanding the relationship between the augmentation of GMax muscle excitation induced by the barbell hip thrust and horizontal displacement speed suggests that this exercise is crucial for enhancing athletic performance in both vertical and horizontal jumps, as well as in running movements (Williams et al., 2021; Bartolomei et al., 2024).

Regarding GMax hypertrophy, Plotkin et al. (2023) noted that the barbell back squat and barbell hip thrust elicit an uneven hypertrophic response within the GMax. Specifically, both exercises result in more pronounced increases in the cross-sectional area of the lower fibers of the GMax compared to other evaluated regions. This disparity in muscular response can be attributed to the mechanical characteristics inherent in the movement, as the exercises are predominantly conducted within the sagittal plane. Research involving sprinters and weightlifters suggests that different joint movements can impose greater stress on targeted muscle areas, thereby fostering specific morphological adaptations (Kenan and Işık, 2021; Takahashi et al., 2022). Consequently, it is conceivable that exercises performed in the sagittal plane, such as squats, leg presses, and hip thrusts, may induce more substantial alterations in the lower fibers of the GMax compared to other regions. Given the efficacy of the barbell hip thrust as an exercise for enhancing neuromuscular performance in both acute (Gautam et al., 2024) and chronic (Sánchez-Sabaté et al., 2024) contexts of high-speed horizontal vector force sports, and recognizing that GMax volume is correlated with performance metrics in sprinters (Miller et al., 2021; Takahashi et al., 2022), chronic training involving barbell hip thrusts may further augment outcomes associated with athletic performance. Furthermore, Sánchez-Sabaté et al. (2024) argue that the specificity of the force vector in physical activity should be of paramount importance when selecting exercises for targeted strength training. Finally, Takahashi et al. (2025) found that the specific torque for hip extension and external rotation was generally greater in the distal region than in other areas. In contrast, for hip abduction, it appeared to be more pronounced in the proximal region compared to the other regions. Therefore, it is plausible to select specific exercises that aim to increase torque in particular regions of the GMax.

Among the numerous technical adaptations of hip thrusts, the original and American variations are particularly noteworthy (Contreras et al., 2016). Both variations have been demonstrated to possess similar effectiveness in stimulating the GMax (Contreras et al., 2016). However, in recent years, these variations have undergone technical modifications designed to enhance comfort and performance, resulting in the emergence of the hinge and scoop variations. The primary objective of the hinge variation is to elevate the hips to their apex position at the end of each repetition, thereby achieving hip hyperextension within the designated ROM (Contreras et al., 2011). Conversely, the scoop variation effectively mitigates lumbar hyperextension during the lockout phase, which is also referred to as the hip thrust with posterior pelvic tilt (Contreras et al., 2016). Concerning GMax hypertrophy, both variations appear to exhibit substantial effectiveness (Plotkin et al., 2023; Kassiano et al., 2024).

Hip extension exercises incorporating elastic bands, ankle weights, and various resistance profiles are commonly integrated into GMax training protocols. Short et al. (2021) conducted a study to evaluate the effects of the kneeling hip extension exercise performed with bands of different tensions over a 6-week training duration. The authors concluded that this exercise significantly enhanced the thickness of the upper fibers of the GMax. Furthermore, the researchers examined the hypothesis that training with blood flow restriction (BFR) applied to the proximal thigh could result in more substantial improvements in neighboring muscles, such as the GMax. Following the training regime, they observed more clinically significant gains in GMax hypertrophy associated with BFR training.

In support of the efficacy of single-joint exercises, Maeo et al. (2023) presented their findings at the 28th Annual Congress of the European College of Sport Science, demonstrating a significant increase in GMax volume following training with the “apolete” exercise. Furthermore, they demonstrated that training with the apolete exercise, utilizing a ROM from 90 to 45° and at an elongated muscle length, resulted in more pronounced increases in GMax compared to traditional training using a full ROM from 90 to 0°.

The evidence suggests that both single-joint and multi-joint exercises, when executed individually, significantly contribute to the hypertrophy of the GMax. Nonetheless, this systematic review highlights that integrating diverse exercise modalities can be an effective strategy for promoting GMax muscle hypertrophy.

Barbalho et al. (2021) conducted a study investigating the effects of various exercise combinations on GMax thickness. Participants were stratified into three groups: one group that performed exclusively multi-joint exercises (MJ), such as back squats, deadlifts, lunges, and leg presses; another group that incorporated both multi-joint and single-joint exercises (MSJ), including back squats, barbell hip thrusts, and kneeling kickbacks; and a final group that engaged solely in single-joint exercises (SJ), which encompassed barbell hip thrusts and kneeling kickbacks.

After 6 months of training, the MJ and MSJ groups exhibited comparable increases in GMax thickness. However, it is crucial to note that the MJ group completed a greater volume of sets for the hip extensor muscles, with 12 sets compared to 8 for the MSJ group and 5 for the SJ group. This observation suggests that training volume should be taken into account when evaluating the efficacy of each exercise type. Furthermore, the findings suggest that combining multi-joint and single-joint exercises with fewer sets, as implemented in the MSJ group, can yield similar gains in GMax relative to a training regimen predominantly featuring multi-joint exercises. Therefore, the selection of exercise combinations for GMax development should be guided by individual muscle requirements or deficiencies.

Recent evidence indicates that the barbell hip thrust exercise, when combined with other movements, can positively influence the growth of the GMax muscle (Barbalho et al., 2021; Kassiano et al., 2024). The study conducted by Kassiano et al. (2024) demonstrated that incorporating the barbell hip thrust into a training program alongside exercises like the incline leg press and the Romanian deadlift (though referred to in the article as the stiff-leg deadlift) resulted in a more significant increase in GMax thickness compared to a program that excluded the barbell hip thrust. This finding suggests that specific hip extension exercises, such as the barbell hip thrust, primarily promote specific and targeted gains in the GMax. Therefore, it is considered one of the best exercise options for achieving significant hypertrophy of the GMax.

A recent study conducted by Bartolomei et al. (2024) investigated the effects of two distinct training protocols on GMax hypertrophy. The vertical training (VT) group primarily concentrated on the back squat, step-up exercises, and box jumps. Conversely, the horizontal training (HT) group participated in exercises that included barbell hip thrusts, reverse hyperextensions, and long jumps. Following a 6-week duration, the authors concluded that both training protocols substantially contributed to enhancements in GMax thickness. This finding implies that there is no singular method for stimulating GMax growth; instead, it recognizes that various exercises can be utilized for effective practical training. Furthermore, the authors emphasize that the selection of training protocol should consider the primary outcomes and objectives pertinent to each patient, practitioner, or athlete. Notably, although the HT group showed significant improvements in horizontal jumps and GMax hypertrophy, this was not reflected in the vastus medialis. In contrast, both groups, HT and VT, demonstrated enhancements in the vertical jump.

It is imperative to underscore that multi-joint exercises, such as squat variations, leg presses, and step-ups, not only facilitate hypertrophy of the GMax but also the quadriceps femoris muscles (Trindade et al., 2019; Plotkin et al., 2023; Bartolomei et al., 2024). Consequently, exercises that emphasize primary hip extension, including barbell hip thrusts and reverse hip hyperextensions, are recommended when the objective is to optimize GMax hypertrophy while minimizing stress on adjacent muscles (Plotkin et al., 2023; Bartolomei et al., 2024). Conversely, incorporating exercises such as squat variations and hip thrusts into a training regimen can enhance development in the GMax alongside other muscle groups, should trainers and practitioners seek to improve overall physique. This consideration is particularly pertinent, considering the highest torque and muscle activation points that various exercise modalities produce.

In our meta-analysis, GMax hypertrophy demonstrated a moderate effect [SMD 0.71, 95% CI (0.50, 0.91), p < 0.00001] and a low level of heterogeneity (p = 0.17, I^2^ = 22%). Conversely, the subgroup analysis pertaining to the type of hypertrophic outcome indicates a large effect for muscle volume [three studies, SMD 0.95, 95% CI (0.33, 1.57), p = 0.003, I^2^ = 67%] and cross-sectional area [one study, SMD 0.93, 95% CI (0.43, 1.43), p = 0.0003, I^2^ = 0%], as well as a moderate effect for morphological assessment via muscle thickness [seven studies, SMD 0.61, 95% CI (0.38, 0.84), p < 0.00001, I^2^ = 0%]. According to Vigotsky et al. (2018), various morphological assessment methods and measurement sites significantly impact the outcomes of muscle hypertrophy, as well as the correlation between hypertrophy and gains in muscle strength. The techniques used for measurement and analysis greatly impact the conclusions drawn from a given dataset. Analyses that overlook inter-individual differences may underestimate the relationship between hypertrophy and strength gains. Employing diverse methods to assess muscle size can lead to varying results. Future studies should employ robust experimental designs and analytical techniques that account for various mechanistic sources of strength gain and individual differences. Furthermore, regarding the subgroup analysis of single [seven studies, SMD 0.74, 95% CI (0.36, 1.13), p = 0.0001, I^2^ = 52%] and combined [six studies, SMD 0.68, 95% CI (0.44, 0.92), p < 0.00001, I^2^ = 0%] training protocols, moderate effects were demonstrated. The moderate degree of heterogeneity observed in studies involving single exercises may also be attributed to the differing sample cohorts used, as well as varying training protocols and morphological assessment measures. Additionally, the considerable heterogeneity observed in the muscle volume outcome (I^2^ = 67%) and the single exercise outcome (I^2^ = 52%) appears to be influenced by the larger effect size reported by Popov et al. (2006).

Training status may be a characteristic that influences the study’s outcome. However, our subgroup analysis indicated that the effect size was moderate for both untrained [eight studies, SMD 0.74, 95%CI (0.47, 1.01), p < 0.00001, I2 = 38%] and trained [three studies, SMD 0.64, 95%CI (0.30, 0.98), p = 0.0002, I2 = 0%] individuals. Therefore, the different training statuses—untrained and trained—do not appear to affect the GMax hypertrophic responses directly.

In our analysis of the potential risk of bias and methodological quality, we identified several factors that may have influenced the interpretation and analysis of results in some of the studies included. These factors included the absence of technical descriptions for the exercises, failure to quantify the prior volume of sets in studies involving experienced individuals, lack of sample size calculations, insufficient randomization strategies, and inadequate blinding concealment, among other issues.

For instance, in the study by Barbalho et al. (2020), the authors did not provide images illustrating how they measured the thickness of the GMax, nor did they detail the previous GMax training volumes or the training technique used for the Barbell hip thrust exercise. The only information regarding the ROM for this exercise is found in the fourth paragraph of the discussion section, where the authors mention that participants trained with a ROM of 45°. This indicates a partial movement and a shorter length. In contrast, Brazil et al. (2021) suggest that the optimal ROM for the Barbell hip thrust exercise is approximately 75° ± 19°.

Although the authors reported significant muscle gains of 3.7%, these results are likely a consequence of the limited training range and do not reflect the ideal ROM of the exercise (approximately 75° ± 19°). Furthermore, the reported hypertrophy gains in the GMax group for the back squat are questionable. The authors failed to mention the training volume before measuring GMax thickness, and the 10% gain they observed, alongside the reported 39.5% strength gains in the full-back squat, raises serious doubts about their findings. Given that the participants in this study were women with an average of 5 years of experience in strength training, it is reasonable to expect that they would have been training with various exercises and multiple weekly sets targeting the GMax. Therefore, a reduction or maintenance of GMax thickness would be anticipated rather than a significant increase, primarily because the volunteers in this study performed only one exercise with six weekly sets.

It is also important to note that in another study by the same research group (Barbalho et al., 2021), the absence of appropriate technical descriptions or images for each exercise further complicates the interpretation and dissemination of the findings, as well as the methodological reproduction of their results.

A recent study highlights the significant impact of interpreting magnetic resonance imaging (MRI) images on quantifying results (Shiotani et al., 2024). Specifically, analyzing the GMax while the subject is supine may introduce bias and lead to potential misinterpretation of the findings. In a study by Balshaw et al. (2023), the authors examined the GMax in this position, which could have reduced muscle volume, ultimately affecting the study’s results. However, the authors conducted both pre- and post-assessments under the same conditions. Therefore, the observed changes may still be valid. We urge future researchers to exercise caution when preparing volunteers for analysis and to adhere to best practices in diagnostic analysis.

In a recent publication, the authors highlighted a significant lack of methodological rigor in sports science studies (Preobrazenski et al., 2024). They noted that most studies fail to report the randomization methods used for group assignments and the blinding procedures for analyzing results. Additionally, many studies do not adequately describe the sample size calculations performed, which makes it challenging to apply the results to a larger population. Furthermore, since this research focuses on specific anatomical regions of the GMax, it is essential to establish a standard for analyzing the effects of different planes and axes of movement on the various areas of the GMax, particularly the upper (cranial) and lower (caudal) fibers.

Future research should prioritize enhancing methodological management to ensure more rigorous and reliable results. Additionally, exploring a wider variety of exercise variations would be beneficial. This can include different types of lunges, such as forward, reverse, lateral lunges, Step-up variations, and Bulgarian split squats. Researchers should also examine the hip abduction exercises using different hip flexion positions and various deadlift styles, including sumo and Romanian deadlifts.

Furthermore, incorporating single-joint movements—such as reverse hyperextension and Romanian deadlifts—into studies could provide valuable insights into muscle-specific training outcomes. Finally, researchers should consider examining movements in the coronal and transverse planes, as these are essential for understanding how various exercises affect overall athletic performance, functional fitness, and GMax morphology.

Finally, it is essential to note that the findings of this systematic review with meta-analysis may have been influenced by several factors, including the lack of pre-training history in studies involving experienced individuals, the wide ROM variability across exercises, and differences in study quality. Additionally, including studies with varying training statuses (trained vs. untrained participants) may yield different hypertrophic responses, along with the variability in measurement protocols across studies.

## Conclusion

This study concludes that a diverse range of exercises, irrespective of their focus on a specific joint (single-joint) or multiple joints (multi-joint), and irrespective of whether they are performed independently or in combination, can effectively stimulate the growth of the GMax. The findings suggest that incorporating both types of exercises into a training regimen may enhance muscle development.

## Data Availability

The original contributions presented in the study are included in the article/supplementary material, further inquiries can be directed to the corresponding author.
